# *Candida albicans* Suppresses Nitric Oxide Generation from Macrophages via a Secreted Molecule

**DOI:** 10.1371/journal.pone.0096203

**Published:** 2014-04-22

**Authors:** John R. Collette, Huaijin Zhou, Michael C. Lorenz

**Affiliations:** Department of Microbiology and Molecular Genetics, The University of Texas Health Science Center at Houston, Houston, Texas, United States of America; New Jersey Medical School, Rutgers University, United States of America

## Abstract

Macrophages and neutrophils generate a potent burst of reactive oxygen and nitrogen species as a key aspect of the antimicrobial response. While most successful pathogens, including the fungus *Candida albicans*, encode enzymes for the detoxification of these compounds and repair of the resulting cellular damage, some species actively modulate immune function to suppress the generation of these toxic compounds. We report here that *C. albicans* actively inhibits macrophage production of nitric oxide (NO). NO production was blocked in a dose-dependent manner when live *C. albicans* were incubated with either cultured or bone marrow-derived mouse macrophages. While filamentous growth is a key virulence trait, yeast-locked fungal cells were still capable of dose-dependent NO suppression. *C. albicans* suppresses NO production from macrophages stimulated by exposure to IFN-γ and LPS or cells of the non-pathogenic *Saccharomyces cerevisiae*. The NO inhibitory activity was produced only when the fungal cells were in direct contact with macrophages, but the compound itself was secreted into the culture media. LPS/IFNγ stimulated macrophages cultured in cell-free conditioned media from co-cultures showed reduced levels of iNOS enzymatic activity and lower amounts of iNOS protein. Initial biochemical characterization of this activity indicates that the inhibitor is a small, aqueous, heat-stable compound. In summary, *C. albicans* actively blocks NO production by macrophages via a secreted mediator; these findings expand our understanding of phagocyte modulation by this important fungal pathogen and represent a potential target for intervention to enhance antifungal immune responses.

## Introduction

*Candida albicans* is a commensal fungus residing on the skin and in the oral cavity, gastrointestinal tract, and vagina of the human host. C. albicans cause a variety of superficial infections in individuals with certain risk factors, including oral thrush, vulvovaginal candidiasis, and various cutaneous infections. People with a significant immunodeficiency are more vulnerable to disseminated infection, which causes severe disease frequently resulting in death. The various defenses of the human innate immune system are primarily responsible for prevention of disseminated candidiasis [1].

Phagocytic cells such as macrophages and neutrophils are important mediators of innate immunity, initiating a robust antimicrobial response upon recognition and internalization of potential pathogens. Activation of macrophages and neutrophils results in the synthesis of antimicrobial effectors, including reactive oxygen species (ROS) and reactive nitrogen species (RNS), the latter generated by the inducible nitric oxide synthase (iNOS, NOS2). SCID mice are more susceptible to mucosal *Candida* infections upon iNOS inhibition and macrophages from these mice had a reduced candidacidal activity upon treatment with an iNOS inhibitor in vitro [2]. However, other studies have shown that NO production may not be required to prevent *Candida* infections [3]–[5], and the exact role of NO in antifungal defense remains unresolved.

Upon NO exposure, *C. albicans* initiates a significant transcriptional response including the upregulation of the NO-detoxifying flavohemoglobin gene *YHB1* [6], [7]. Two additional genes, *YHB4* and *YHB5*, also encode flavohemoglobins suggesting that *C. albicans* considers NO a significant enough danger to create and maintain such a detoxifying system. Strains lacking *YHB1*, but not *YHB4* or *YHB5*, were NO hypersensitive in vitro, but had only a modest virulence defect in the mouse model of disseminated hematogenous candidiasis [6], [7] suggesting that the additional flavohemoglobin genes might also be required for the establishment of an infection and/or that NO plays a limited role in antifungal defenses.

*C. albicans* also modulates other macrophage responses. Phagocytosed cells appear to follow a non-classical endocytic pathway [8], though the exact route and mechanism remain unclear. They also actively modulate the pH of the phagolysosome, potentially through the excretion of ammonia generated from amino acid catabolism, creating a neutral pH compartment conducive to hyphal morphogenesis [9], [10]. Secreted superoxide dismutases (SODs) of *C. albicans* detoxify extracellular ROS species: deletion of the *SOD4* and *SOD5* genes results in extensive ROS accumulation and reduced viability during co-culture with macrophages [11]. In addition, *C. albicans* can block NO production from phagocytic cells [12], [13], however the identification of the NO-inhibitory activity and its mechanism of action have not yet been determined.

In this report, we demonstrate that *C. albicans* produces an NO-inhibitory activity upon culturing with primary (bone marrow-derived) and tissue culture macrophages. This NO-inhibitory activity is secreted into co-culture supernatants and requires direct contact between fungal cells and macrophages. The effects on NO production are likely mediated by a failure to fully induce iNOS upon stimulation in the presence of this inhibitor, which preliminary characterization indicates is a small, aqueous, heat-stable compound. These findings represent another layer of modulation of phagocyte function by this important pathogen.

## Methods

### Strains and media

*C. albicans* strains used in this study have been previously described and include the prototrophic wild-type strain SC5314, the non-filamentous *cph1Δ efg1Δ* double mutant HLC54, the *arg1Δ* mutant JRC12, and the *arg1*Δ + *ARG1* complemented strain JRC29 [10], [14]–[16]. The *arg4Δ* (SN152) and *arg5,6Δ* (CNC44) strains were kindly provided by S. Noble and J. Pla [17], [18]. The *S. cerevisiae* strains EM93 and BY4741 have also been described [19]–[21]. Fungal strains were propagated in YPD (1% yeast extract, 2% peptone, and 2% glucose) under standard conditions [22]. RAW264.7 and J774A.1 macrophages (ATCC) were cultured at 37°C with 5% CO_2_ in RPMI culture media supplemented with 10% fetal bovine serum (FBS), 100U/mL penicillin and 100µg/mL streptomycin (Pen-Strep).

Bone marrow-derived macrophages (BMDMs) were isolated from sacrificed ICR mice as described previously [23] and cultured at 37°C with 5% CO_2_ in Iscove's modified Dulbecco's medium (IMDM) supplemented with 10% FBS, Pen-Strep, and 10 ng/mL mouse granulocyte-macrophage colony-stimulating factor (GM-CSF). All animal work was performed in accordance with protocols approved by the Animal Welfare Committee of the University of Texas Health Science Center at Houston.

### Fungal:macrophage co-cultures and NO determination

Nitric oxide (NO) was assayed in supernatants of macrophage-*C. albicans* cocultures in a 24-well plate format using the Greiss reagent to detect nitrite, the product of spontaneous degradation of NO. Macrophages were collected and counted using a hemocytometer and 3×10^5^ cells were seeded into wells of a 24-well plate at least 2 hours prior to the initiation of co-culture with fungi. *C. albicans* or *S. cerevisiae* were grown overnight in YPD, diluted 1:100 in fresh YPD, and grown for up to 4 hours at 30°C to allow cells to reach exponential growth. Cells were pelleted, resuspended in phosphate-buffered saline (PBS), counted, and incubated with macrophages at the indicated ratios. One hour following addition of the fungi, co-cultures were treated with LPS (100 ng/mL) and IFNγ (100U/mL) and incubated for ∼24 hours at 37°C/5% CO_2_. Nitrite was quantified using the Greiss Reagent following the manufacturer's protocol (Molecular Probes).

To assess the role of *C. albicans* viability on NO production by macrophages, exponentially growing fungal cells were heat-killed via incubation at 65°C for 20 minutes prior to co-culturing with macrophages. The requirement for direct contact was assayed by measuring NO production from RAW264.7 macrophages cultured in wells of a 24-well plate with fungal cells present either in the same chamber or separated by a membrane in Transwell inserts (0.4µm polycarbonate membrane, Corning Inc.).

### Collection and manipulation of co-culture supernatants

Culture media was collected from LPS/IFNγ-treated *C. albicans*:RAW264.7 macrophage co-cultures, centrifuged to pellet any cellular debris/dead cells, and supernatant sterilized by passage through a 0.2 µm nylon syringe filter. To assess the ability of this conditioned media to inhibit NO production, naïve macrophages were cultured in a 1:1 mixture of the sterilized supernatant and fresh media for at least 2 hours, then stimulated with LPS and IFNγ.

Conditioned media from *C. albicans*:macrophage co-cultures were size-fractionated using Centricon centrifugal filtration devices with a 3 kDa molecular weight cutoff (MWCO) membrane, separating the supernatant into flow-through (less than 3 kDa) and retained (over 3 kDa) fractions. These samples were dried, resuspended in water, and added to naïve RAW264.7 macrophages stimulated with LPS/IFNγ. Separately, conditioned media was also mixed 1:1 with chloroform to generate aqueous and organic phases. Aqueous and organic layers were separated, dried, resuspended in water, and added to macrophages stimulated with LPS/IFNγ. In addition, macrophages were incubated with conditioned media that had been boiled for 20 minutes at 100°C. For all conditions, nitrite concentrations in the culture media were assayed after 24 hours.

Stability of nitrite supernatant over time was determined by assaying nitrite levels in sterilized co-culture conditioned media for 1–24 hours using the Greiss reagent. Separately, the NO donor DETA-Nonoate (Cayman Chemical) was added to fresh cell-free media and its concentration was assayed over 24 hours.

### Macrophage lysates and Western blotting

2×10^6^ RAW264.7 macrophages were seeded into wells of a 12-well plate for at least 2 hours prior to the initiation of LPS/IFNγ stimulation. Immediately prior to the addition of LPS/IFNγ, cells were washed with PBS and incubated with either fresh RPMI or undiluted co-culture supernatant. Following 8 hours of LPS/IFNγ stimulation, cells were washed with PBS and collected by scraping. Cell pellets were resuspended in RIPA buffer (25 mM TRIS-HCl pH 7.6, 150 mM NaCl, 1% NP40, 1% sodium deoxycholate, 0.1% SDS) and incubated on ice for 5 minutes. Lysates were centrifuged at 14,000×g for 10 minutes at 4°C after which supernatants were transferred to new tubes. Protein concentrations of cell lysates were determined using the Bio-Rad Protein Assay Dye Reagent, and 20 µg of total cellular protein per sample was loaded onto an 8% SDS-polyacrylamide gel. Blots were probed with iNOS antiserum (Santa Cruz Biotechnologies) at a 1:500 dilution followed by HRP-conjugated anti-rabbit IgG antiserum at a 1:3000 dilution. As a control for equivalent protein loading, membranes were also probed with anti-tubulin antiserum (a gift from Dr. Eric Wagner, UT-Houston).

### iNOS activity assays

Intracellular NO production was measured via the fluorimetric Nitric Oxide Synthase Detection System (Sigma-Aldrich), which is based on the iNOS-dependent conversion of a cell-permeable diacetate derivative of 4,5-diamino-fluorescein (DAF-2 DA) to the membrane- impermeant fluorescent molecule triazolofluorescein (DAF-2T) via a 4,5-diamino-fluorescein (DAF2) intermediate. Fluorescence of DAF-2T was measured using a Synergy MX multimode plate reader (Bio-Tek, Winooski, VT) with an excitation wavelength of 490 nm and an emission wavelength of 520 nm.

RAW264.7 macrophages were seeded into wells of a black-walled, clear bottom 96-well tissue culture plates and incubated for 2 hours to allow for adherence. Macrophages were washed with PBS and stimulated with LPS/IFNγ in either RPMI media or co-culture supernatant for ∼24 hours before initiating the iNOS activity assay according to the manufacturer's protocol.

## Results

### *C. albicans* actively suppresses NO production from macrophages

**Figure 1:**
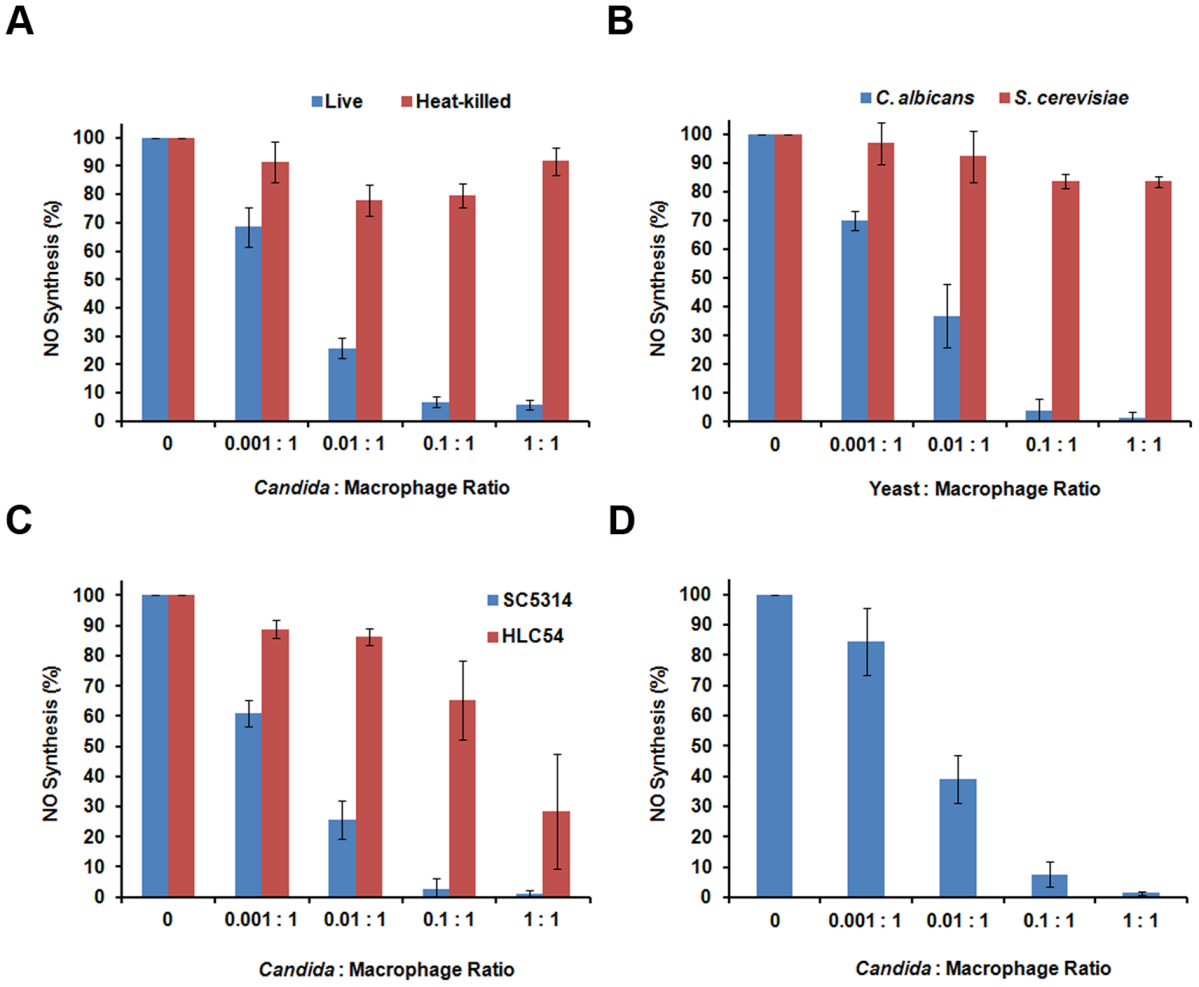
*Candida albicans* actively inhibits nitric oxide (NO) synthesis by macrophages. A. Exponentially growing *C. albicans* cells were mock-treated (Live) or heat-killed prior to co-culturing with LPS/IFNγ-stimulated RAW264.7 mouse macrophages at the indicated *C. albicans*:macrophage ratio. B. Exponentially growing *C. albicans* or *S. cerevisiae* cells were co-cultured with RAW264.7 macrophages at the indicated ratios as in panel A. C. Exponentially growing wildtype (SC5314) or the non-filamentous *cph1Δ efg1Δ* mutant (HLC54) *C. albicans* cells were co-cultured with J774A.1 mouse macrophages as in panel A. D. Exponentially growing *C. albicans* cells were co-cultured with mouse bone marrow-derived mouse macrophages as in panel A. For all panels, supernatants were collected following ∼24 hours of co-culture, and nitrite levels were determined using the Greiss Reagent.

Nitric oxide (NO) is a key component of the antimicrobial burst of phagocytic cells. To assess whether *C. albicans* affects NO production, RAW264.7 mouse macrophages were stimulated with lipopolysaccharide (LPS) and interferon-γ (IFNγ) to upregulate iNOS [24] and co-cultured with increasing amounts of *C. albicans* strain SC5314 for 24 hours. NO is rapidly oxidized in aqueous solutions to nitrite, which was detected in co-culture supernatants via the Greiss reagent. When increasing numbers of fungal cells are co-cultured with a fixed number of macrophages, a corresponding decrease in the amount of NO produced is observed (Figure 1A). This phenomenon is quite robust as a ratio of one *C. albicans* cell per 100 macrophages (0.01:1) resulted in a ∼75% reduction in NO production, and an almost complete block was observed at fungal cell:macrophage ratios of 0.1:1. To determine if live fungal cells were necessary for this NO suppression, LPS/IFNγ-stimulated RAW264.7 macrophages were co-cultured with heat-killed fungal cells and assessed for NO production. As shown in Figure 1A, inviable *C. albicans* cells reduced NO production by only ∼10–20% regardless of the fungal cell:macrophage ratio. This is in sharp contrast to the dose-dependent suppression obtained by live *C. albicans* cells and strongly supports the notion that NO inhibition by *C. albicans* is an active process requiring fungal viability.

To determine whether the NO inhibitory activity is specific to *C. albicans*, LPS/IFNγ-stimulated RAW264.7 macrophages were also co-cultured with the non-pathogenic *Saccharomyces cerevisiae* EM93 strain, which we have previously used in similar studies because it grows well at 37°C [20]. *S. cerevisiae*:macrophage co-cultures did not display a significant block in NO production (∼10–20%) even when fungal cells and macrophages were co-cultured at a 1:1 ratio (Figure 1B).

Following phagocytosis by macrophages, *C. albicans* fungal cells will initiate hyphal growth to facilitate escape from acidic phagosomes and then ultimately escape from the phagocyte. To address whether or not the reduction in macrophage NO production by *C. albicans* is due to extensive macrophage lysis during the course of these experiments, we utilized the non-filamentous mutant *C. albicans* strain HLC54 [16] in co-cultures with a second murine macrophage-like cell line, J774A.1. Dose-dependent suppression of NO production was observed with these non-filamentous fungal cells (Figure 1C), however greater numbers of HLC54 cells were required to observe this suppression as compared to the hyphal-competent SC5314 strain. We conclude from this experiment that hyphal morphogenesis is not required for this phenomenon, but that it contributes to NO inhibition, potentially because of increased macrophage damage.

The experiments in Figs. 1A–B used RAW264.7 cells while those in Fig. 1C used J774A.1 cells. These cell line are the most common used for in vitro studies with *C. albicans*, but both have alterations that may affect fungal recognition. RAW264.7 cells, for instance, lack the ASC component of the inflammasome while J774A.1 cells do not express the mannose receptor [25]. The ability of wild-type *C. albicans* to inhibit NO production is essentially the same in both cell lines (Fig. 1A–C). We also investigated whether NO inhibition by *C. albicans* is a phenomenon observed in primary macrophages. Mouse bone marrow-derived macrophages (BMDMs) were stimulated with LPS/IFNγ and co-cultured with increasing amounts of *C. albicans*. Thus, *C. albicans* suppresses NO production from two different cell lines as well as primary cells (Fig. 1), indicating that this is a general phenomenon. For simplicity, cultured macrophages were utilized for the remainder of the experiments.

**Figure 2:**
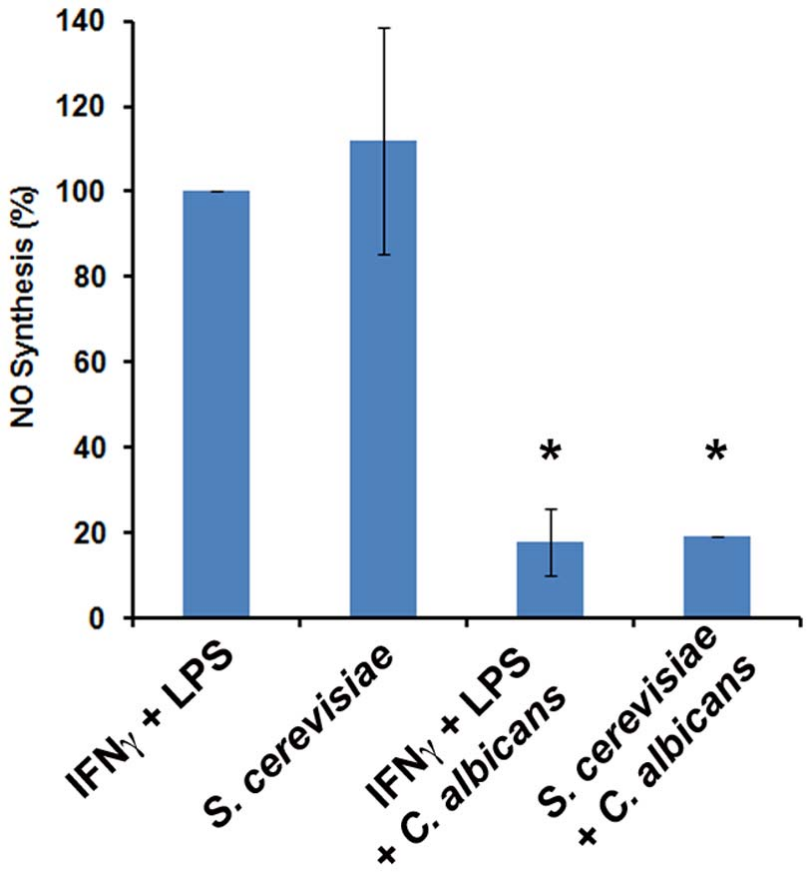
NO inhibition by *C. albicans* in the absence of LPS/IFNγ stimulation. Unstimulated RAW264.7 macrophages were co-cultured with either live *S. cerevisiae* strain BY4741, live *C. albicans* strain HLC54, or both (at a 1:1 ratio). Following ∼24 hours of co-culture, supernatants were collected and nitrite levels assayed via the Greiss Reagent. Nitrite levels are expressed as a percentage of that generated by RAW264.7 macrophages stimulated with LPS/IFNγ for ∼24 hours in the absence of fungi. The ‘*’ indicates a p-value of <0.05 relative to IFNγ/LPS or *S. cerevisiae* alone.

Macrophages activated by LPS/IFNγ stimulation induce robust iNOS expression, but in vivo *C. albicans* recognition by phagocytes is mediated by a variety of receptors that recognize fungal associated molecular patterns, including Dectin-1, Dectin-2, the Mannose Receptor, and several Toll-Like Receptors [1]. To verify that the failure to induce NO production is not due to a lack of recognition of the fungal cells or interference with LPS/IFNγ signaling, we stimulated RAW264.7 macrophages either with IFNγ/LPS or with live cells of *S. cerevisiae* strain BY4741, in the presence or absence of the non-filamentous *C. albicans* strain HLC54 (used to minimize macrophage damage from hyphal growth). As shown in Fig. 2, *S. cerevisiae* cells induced robust NO production, comparable to LPS/IFNγ, indicating that these cells are activated in response to fungal antigens. In contrast, little NO was detected when macrophages were incubated with *S. cerevisiae* in the presence of *C. albicans.* Nearly identical results were obtained using J774A.1 cells. Thus, *C. albicans* inhibits NO synthesis induced by multiple signaling pathways.

### Direct contact between *C. albicans* and macrophages required for full NO suppression

**Figure 3:**
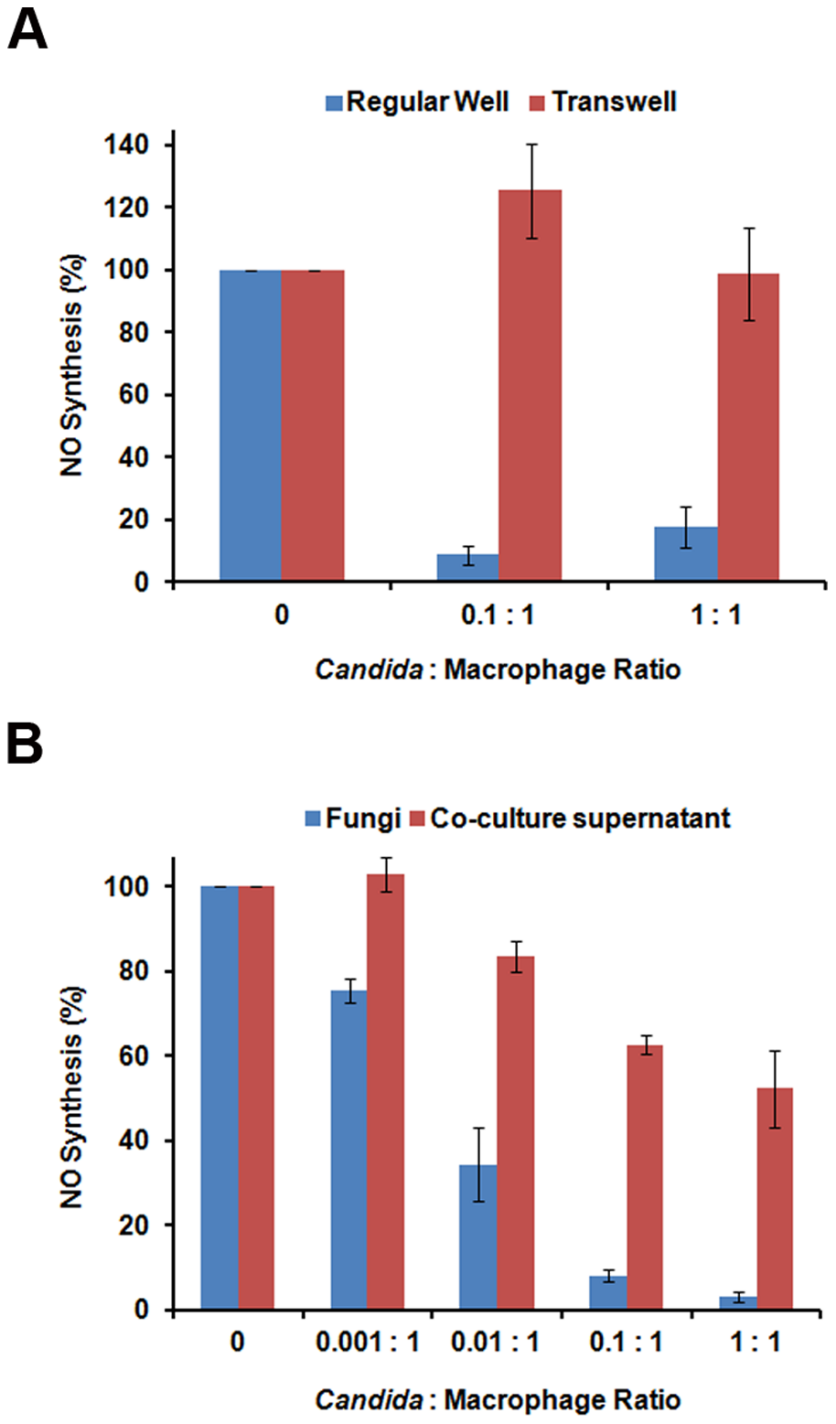
NO inhibition by *C. albicans* requires direct *C. albicans*-macrophage contact and is mediated by a secreted activity. A. *C. albicans* and LPS/IFNγ-stimulated RAW264.7 macrophages were co-cultured on either the same side (Regular Well) or the opposite side (Transwell) of polycarbonate membrane Transwell inserts containing 0.4µm pores. Supernatants were collected following ∼24 hours of co-culture, and nitrite levels were determined using the Greiss Reagent. B. LPS/IFNγ-stimulated RAW264.7 macrophages were cultured with either live *C. albicans* (Fungi) at the indicated ratio or with culture media (co-culture supernatant) previously collected from *C. albicans*: RAW264.7 macrophage co-cultures. Supernatants were collected following ∼24 hours of culture, and nitrite levels were determined using the Greiss Reagent.

Macrophages generate antimicrobials such as NO following recognition and phagocytosis of various pathogenic microbes. The requirement for direct contact between *C. albicans* and macrophages in order to block NO production was assessed by culturing each cell type on opposite sides of a Transwell barrier containing pores too small for the diffusion of whole cells but suitable for the diffusion of small molecules. Nitric oxide levels in co-cultures of LPS/IFNγ-stimulated macrophages and *C. albicans* on the same side of the Transwell were dramatically lower than in control macrophage cultures (Figure 3A, Regular Well). However, when macrophages and fungal cells were cultured on opposite sides of the Transwell barrier NO synthesis was unchanged (Figure 3A, Transwell), indicating that direct contact was necessary for the NO inhibitory activity of *C. albicans*.

### *C. albicans* secretes an NO inhibitory activity

While direct contact between *C. albicans* and macrophages is required for suppression of NO production, this may be due to a cell autonomous secreted molecule produced only upon contact/phagocytosis or through a cell-dependent mechanism. To address this question, we assayed cell-free co-culture conditioned media for suppressive activity. To do this, culture media was collected from *C. albicans*-macrophage co-cultures of LPS/IFNγ-stimulated macrophages and *C. albicans*, filter-sterilized, and mixed 1:1 with fresh RPMI + serum to ensure sufficient nutrients were present to support macrophage growth. In this media, naïve macrophages stimulated with LPS/IFNγ in the absence of fungal cells produced significantly less NO than controls (Figure 3B). The decrease in NO production observed in macrophages cultured with sterilized co-culture supernatant was roughly half than seen with live cells, which is consistent with the 1:1 dilution of the conditioned media. Supernatant taken from monocultures of *C. albicans* or macrophages alone in RPMI + serum did not inhibit NO production from stimulated macrophages (data not shown). Thus, the inhibitory activity is produced only after direct cell contact but is then secreted into the supernatant where it can act autonomously.

**Figure 4:**
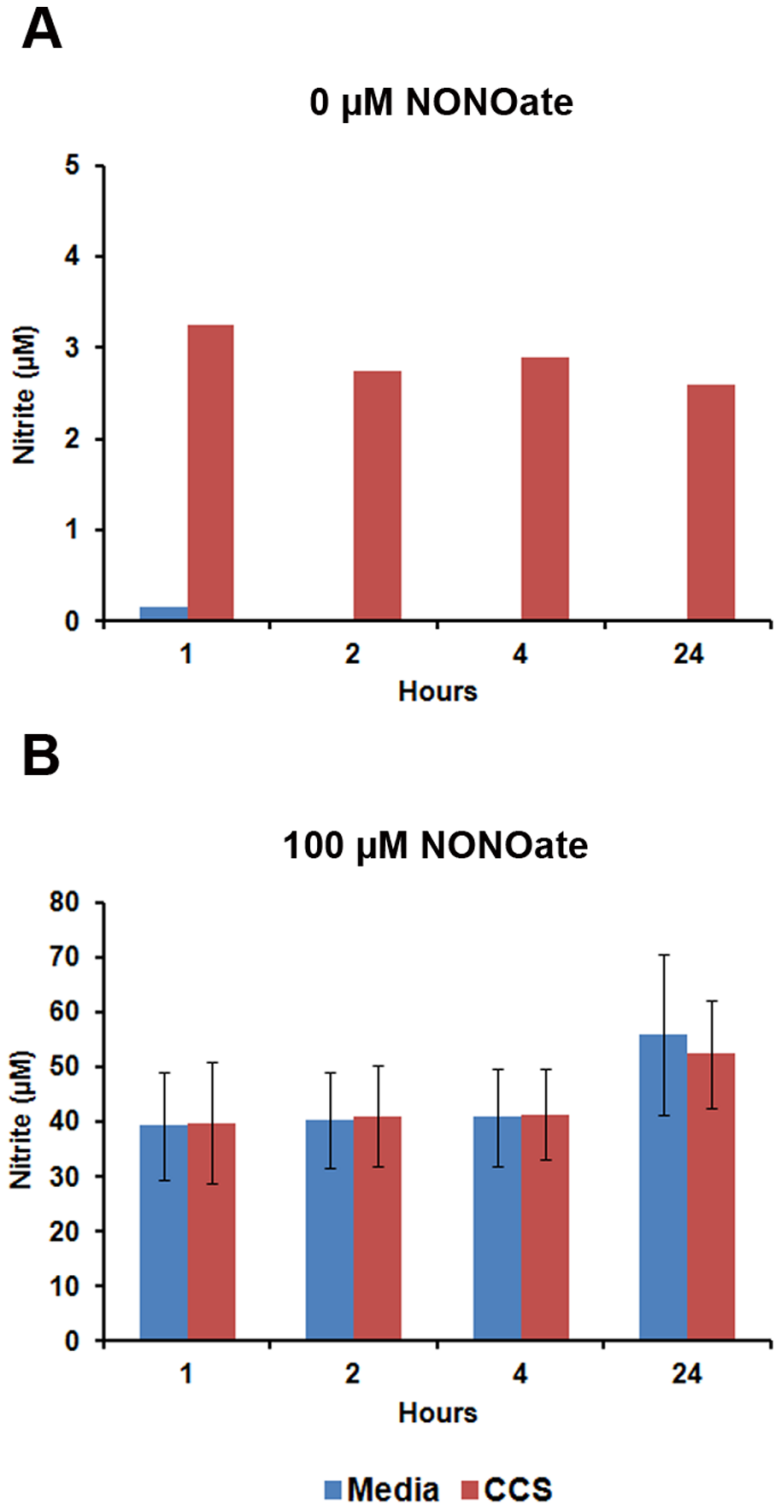
The secreted NO inhibitory activity of *C. albicans* does not sequester NO upon addition of an exogenous NO donor. A. Conditioned media from a 24-culture was filter sterilized and incubated for 24 hours at 37°C. At the indicated times, aliquots were removed and assayed for nitrite concentration. B. 100 µM DETA-NONOate was added to fresh media or filter sterilized conditioned media from a 24 hour co-culture (CCS) and incubated at 37°C. Nitrite concentrations were assayed at the indicated times using the Greiss Reagent.

It remained possible that the secreted activity was not truly blocking NO production by iNOS, but rather was masking the actual amount of nitrite present in the culture media by converting it into a form not detectable by the Greiss reaction. If so, the amount of detectable nitrite in conditioned media should decay over time through the action of the inhibitor. To address this possibility, we collected and sterilized conditioned media from a co-culture and incubated it at 37°C for up to 24 hours. Nitrite concentrations in this media were stable over time (Figure 4A), indicating that the *C. albicans*-derived activity did not affect nitrite once it was generated.

Similarly, it was possible that *C. albicans* directly detoxifies NO into a chemical moiety that cannot be measured. To test this, we incubated either regular RPMI culture media or co-culture supernatant with 100µM diethylenetriamine-NONOate (DETA-NONOate), a nitric oxide donor molecule that rapidly dissociates in aqueous solutions. Aliquots of media or co-culture supernatant were removed at various time-points up to 24 hours following DETA-NONOate addition and nitrite measurements were assessed. Addition of DETA-NONOate to both media and co-culture supernatants resulted in rapid accumulation of nitrite within the first hour, and these nitrite levels remained relatively constant over the course of 24 hours (Figure 4B), suggesting that the active co-culture supernatant is not masking the nitrite signal and is indeed inhibiting NO synthesis.

### NO inhibition is mediated by a small, aqueous, heat-stable compound

**Figure 5:**
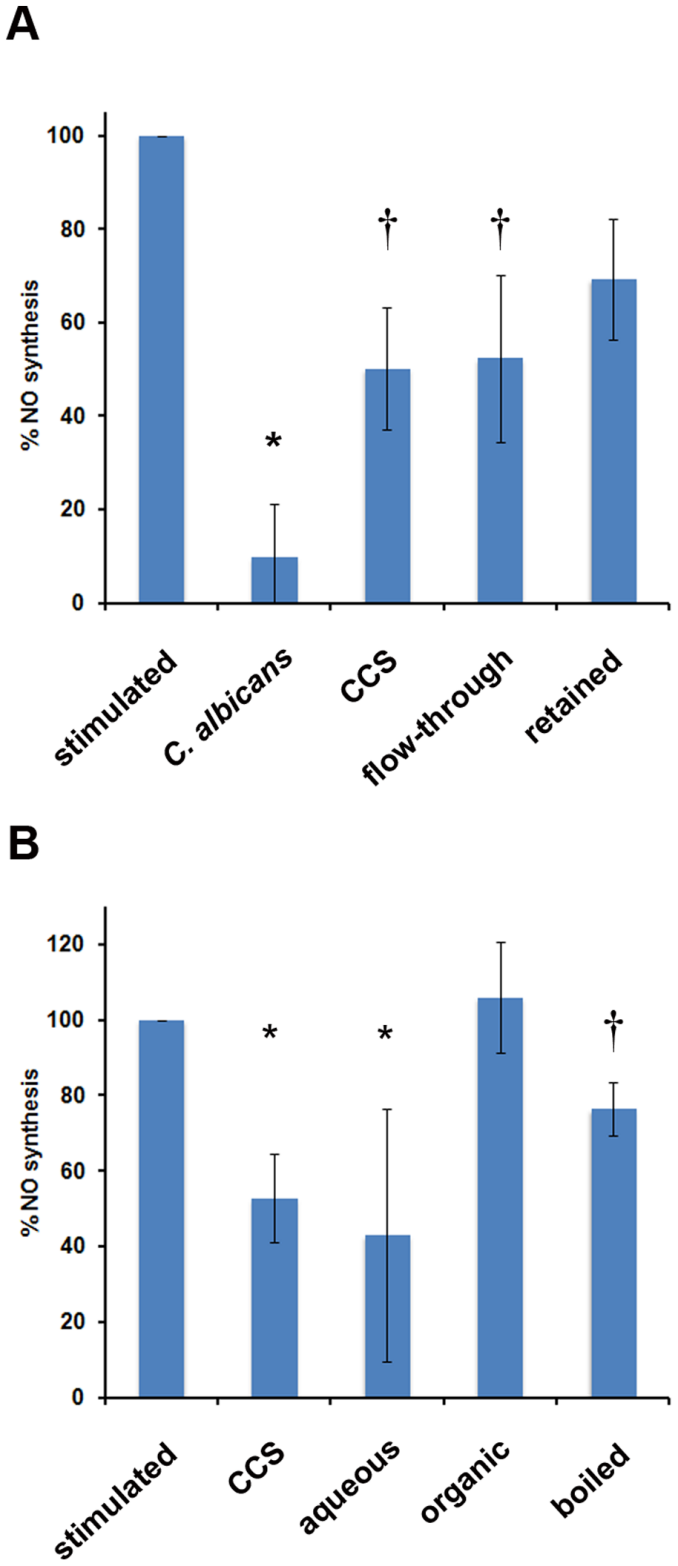
*C. albicans* NO inhibition is mediated by a small, aqueous, and heat-stable activity. A. Conditioned media from *C. albicans*:macrophage co-cultures were size fractionated using Centricon centrifugal filtration devices containing a 3 kDa MWCO, separating the supernatant into flow-through and retained fractions. These samples were dried, resuspended in water, and added to naïve RAW264.7 macrophages stimulated with LPS/IFNγ. Stimulated macrophages, macrophages co-cultured with live *C. albicans*, and the complete co-culture supernatant (CCS) are used as controls. B. Conditioned media was prepared as in panel A and mixed 1:1 with chloroform. The organic and aqueous layers were separated, dried, and resuspended in water, then added to stimulated cultures of naïve macrophages. Separately, stimulated macrophages were incubated in conditioned media that had been boiled for 20 minutes at 100°C. Stimulated macrophages and complete co-culture supernatant (CCS) are used as controls. For both panels A and B, nitrite concentrations in the culture media were determined after 24 hours. Data statistically significant from the control stimulated macrophages (p<0.05) is indicated by ‘*’. In panel A, the ‘†’ indicates data that is not significantly different from the positive (*C. albicans*) or negative (stimulated) controls, likely because of the dilution of the supernatant into fresh media. In panel B, the ‘†’ indicates data intermediate between the stimulated control and the unfractionated co-culture supernatant.

Identification of the secreted compound(s) responsible for this NO suppression by *C. albicans* will be critical for understanding the mechanism of action. The composition of the co-culture supernatant is quite extensive, containing a broad spectrum of molecular species including ions, co-factors, vitamins, carbohydrates, and proteins. Experiments were conducted to discriminate among these numerous possibilities. To obtain information about the molecular weight of the NO inhibitory activity, filter-sterilized conditioned media from *C. albicans*: macrophage co-cultures were size fractionated using centrifugal filtration devices with a 3 kDa MWCO, separating the supernatant into flow-through and retained fractions. Fractions were incubated with LPS/IFNγ-stimulated RAW264.7 macrophages and nitrite concentrations in the culture media were assayed following 24 hours. Nitrite levels in macrophages treated with the flow-through fraction were comparable to the levels observed in macrophages treated with co-culture supernatant (CCS) (Figure 5A), indicating that this NO inhibitory activity passed through the filter and was less than ∼3 kDa in size. A small amount of activity was observed upon addition of the retained fraction (Figure 5A), however the presence of this weaker activity can be attributed to the retention of a small volume of the residual liquid in the upper chamber of the filter following centrifugation.

In separate experiments, conditioned media were mixed with an equivalent amount of chloroform to generate aqueous and organic fractions. These fractions were separated, dried, resuspended in water, and added to LPS/IFNγ-stimuated macrophages. Assay of nitrite levels following 24 hours revealed that stimulated macrophages treated with aqueous fractions still blocked NO production (Figure 5B) whereas treatment with organic fractions had no effect. Similarly, conditioned media boiled for 20 minutes prior to incubation with stimulated macrophages displayed a modest reduction in nitrite levels (Figure 5B). All together, these data suggest that secretion of a small, aqueous, heat-stable compound is responsible for *C. albicans*-mediated NO suppression.

### NO inhibitory activity reduces macrophage iNOS expression

**Figure 6:**
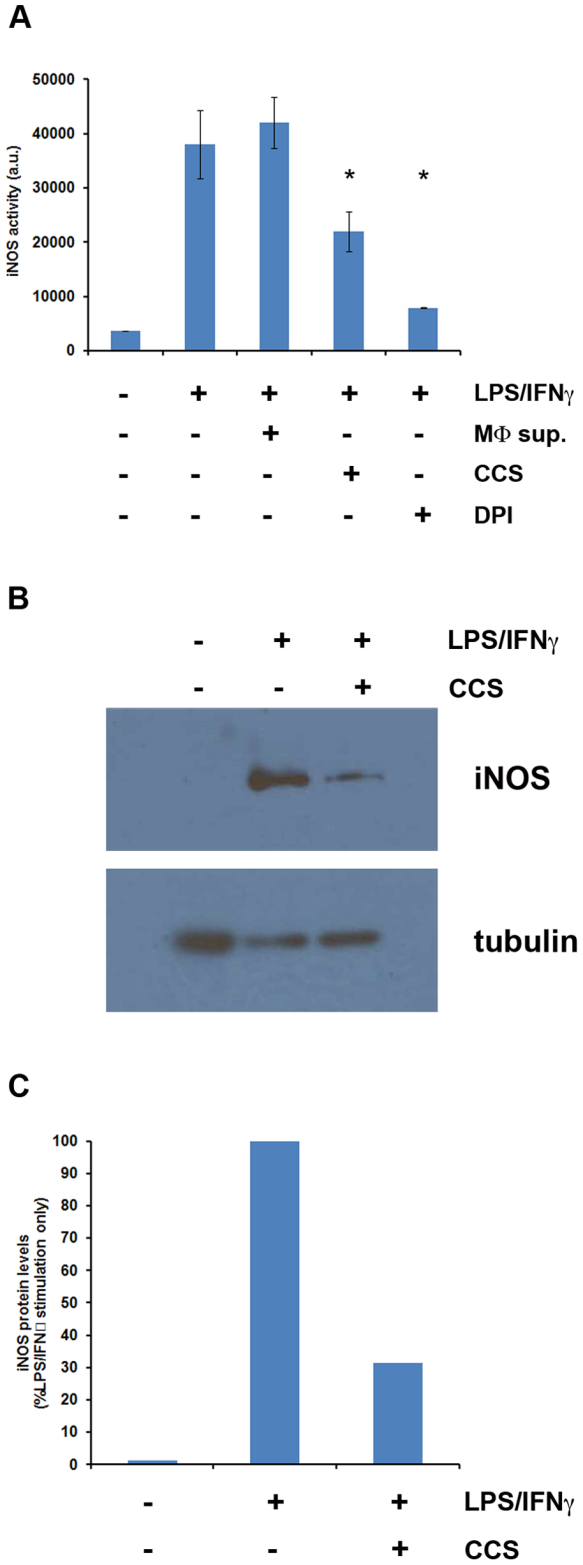
The secreted NO inhibitory activity of *C. albicans* modulates iNOS protein levels. A. Enzyme activity assays were performed using unstimulated and LPS/IFNγ-stimulated RAW264.7 macrophages cultured in RPMI media (LPS/IFNγ), sterilized macrophage supernatant (MΦ sup.), or co-culture supernatant (CCS). Assays were performed in triplicate according to the manufacturer's protocol. During these assays, a known iNOS inhibitor, diphenyleneiodonium chloride (DPI), was utilized as a positive control for enzyme inhibition. The ‘*’ indicates a significant (p<0.05) difference relative to the IFNγ/LPS-stimulated macrophages. B. Western blot analyses of cell lysates prepared from unstimulated macrophages, LPS/IFNγ-stimulated macrophages cultured in normal media, and LPS/IFNγ-stimulated macrophages cultured in undiluted co-culture supernatant (CCS), each for 8 hours. C. Quantitation of iNOS protein levels from panel B. The amount of iNOS protein present following 8 hours of LPS/IFNγ stimulation in normal media was set to 100%.

iNOS (NOS2) is the enzyme responsible for NO production within macrophages and is a potential target for the mechanism of action of the secreted NO inhibitory activity by *C. albicans*. Enzymatic activity of the iNOS protein was measured by following the fate of 4,5-diamino-fluorescein diacetate (DAF-2 DA), a cell-permeable compound that is converted to the impermeant fluorescent molecule triazolofluorescein (DAF-2T) via an iNOS-dependent mechanism. LPS/IFNγ-stimulated macrophages were cultured in either RPMI media or co-culture supernatant for 24 hours prior to the addition of DAF-2 DA. Stimulated macrophages cultured in RPMI displayed a strong fluorescence indicative of robust iNOS enzymatic activity (Figure 6A), consistent with previously published results for these conditions [24]. Macrophages cultured in co-culture supernatant (CCS) showed significantly reduced fluorescence readings indicative of reduced iNOS activity which was not observed when macrophage supernatant (MΦ sup.) was utilized (Figure 6A).

To investigate the possibility that *C. albicans* modulates iNOS activity by altering iNOS protein expression, RAW264.7 macrophages were stimulated with LPS and IFNγ for 8 hours in normal culture media or in undiluted, cell-free co-culture supernatant. Stimulation of RAW264.7 cells with LPS/IFNγ induced expression of the iNOS protein as detected by Western blotting (Figure 6B); iNOS was not detectable in the absence of stimulation. Stimulated macrophages incubated with co-culture supernatant displayed a significant (∼70%) reduction in the levels of iNOS protein (Figure 6C), indicating that the secreted activity of *C. albicans* reduces iNOS induction within macrophages following activation.

The amino acid arginine is the substrate for the iNOS-catalyzed production of NO, and many of the known inhibitors of iNOS are either intermediates of arginine biosynthesis, or their structural analogs. α-difluoromethylornithine (an inhibitor of ornithine decarboxylase), N-iminoethyl-L-ornithine (NIO), and N^G^-N^G^-dimethylarginine (ADMA; synthesized by endothelial cells) have all been shown to inhibit NO production from phagocytes [26]-[28]. Following phagocytosis by macrophages, *C. albicans* significantly upregulates genes of the arginine biosynthetic pathway [23], [29], leading us to examine whether this pathogen was synthesizing an arginine derivative as the inhibitor. LPS/IFNγ-stimulated RAW264.7 macrophages co-cultured with an *arg1Δ* deletion strain also showed a comparable decrease in NO production when compared to the isogenic wildtype SC5314 strain (Figure S1), indicating that the arginine pathway was dispensable for iNOS inhibition. We also found no effect of an *arg3Δ* mutant generated by the SAT-flipper approach in SC5314 or *arg4Δ* or *arg5,6Δ* mutants constructed in the RM1000 strain (data not shown).

## Discussion

In this study, we report that *C. albicans* is capable of blocking nitric oxide (NO) production upon culturing with either tissue culture or bone marrow-derived macrophages. This is independent of filamentous growth of the fungus and occurs in co-cultures with both immortalized and primary bone marrow derived murine macrophages. Incubation of LPS/IFNγ-stimulated macrophages with co-culture supernatant reduces the enzymatic activity and protein levels of iNOS. While the exact composition of this activity remains unknown, our data is most consistent with it being a fungal-derived product and our initial characterization suggests that it is an aqueous, thermoresistant compound less than 3,000 Daltons in size.

Previous studies have reported that *C. albicans* can suppress NO production in macrophages [12], [13], yet with some important differences. Both studies suggested that direct cell contact was not required for inhibition, as they observed a partial or nearly complete reduction in NO generation when cells were separated by a Transwell barrier. In contrast, our Transwell data with RAW264.7 macrophages provides compelling evidence that direct contact between *C. albicans* and cultured macrophages is absolutely required in order to block NO production. We have not seen the inhibitory activity in supernatants of *C. albicans* cultures grown in the absence of macrophages in any of several media tested (data not shown), further suggesting a key role for cell-cell contact.

Differing effects of the *C. albicans* NO inhibitory activity on iNOS have also been previously reported. Schroppel et al. observed a modest decrease in iNOS protein, but not mRNA levels, coupled with a far greater decrease in iNOS enzymatic activity [12], [13]. Here we report that LPS/IFNγ-stimulated RAW264.7 macrophages cultured in co-culture supernatant have significantly lower levels of the iNOS protein and similarly reduced iNOS enzymatic activity, reconciling these observations and strongly suggesting that the affect on NO production comes from a failure to fully induce this protein rather than inhibition of enzymatic activity. Some of these differences are perhaps due to the different types of macrophages used, as the other studies used peritoneal exudate macrophages whereas we have used both cultured lines and bone marrow-derived cells.

Following macrophage phagocytosis, *C. albicans* initiates a robust transcriptional response, including upregulation of nearly all the arginine (*ARG*) biosynthetic pathway genes [23], [29]. As arginine is the substrate for iNOS, we hypothesized that the ARG pathway might be involved in generating a competitive inhibitor of the iNOS enzyme. Deletion mutants of multiple *ARG* genes were tested, and all mutant strains were able to block NO production comparable to the isogenic wildtype strain, suggesting the ARG pathway was dispensable for NO inhibition. In fact, *C. albicans ARG* gene upregulation following macrophage phagocytosis is dependent on the oxidative burst of reactive oxygen species generated by the macrophage NAPDH oxidase [23].

We have not yet been able to purify this inhibitory activity. A large number of compounds present in tissue culture media fit the physical characteristics we have associated with this substance, including amino acids, nucleotides, salts, and vitamins. We have attempted to concentrate the activity through lyophilization, ion exchange chromatography, and reverse phase C18 cartridge purification. In each case, the resulting sample was potently toxic to the macrophages, likely due to very high concentrations of salts or amino acids. We are optimistic that the results presented here will motivate further study of this interesting phenomenon.

Our data is consistent with this inhibitory activity being a product of the fungal cells. However, it is formally possible that contact with *C. albicans* may induce a subset of macrophages to produce an apoptotic, pyroptotic, or other “death” signal that explains the reduced NO production. We do not favor this explanation for three reasons: first, naive macrophages cultured in conditioned media do not show widespread death, as measured by trypan blue exclusion (data not shown). Further, production of reactive oxygen species is unaffected by culturing with either conditioned media or live *C. albicans* cells. Finally, the reduction in iNOS protein levels were measured normalized to tubulin, so there is a marked difference in abundance of this protein relative to a housekeeping protein. Together, these suggest that macrophage death is not the most likely explanation for the phenomenon we observe. It is possible that this inhibitory activity comes from the macrophage in response to viable *C. albicans* cells, though this would be specific to this species, since *S. cerevisiae*, which presents a similar set of pathogen-associated molecular patterns, does not inhibit NO production.

Additional fungal pathogens, including *Cryptococcus neoformans*, *Blastomyces dermatitidis*, *Coccidioides posadasii*, and *Coccidioides immitis* have also been shown to block macrophage production of NO in vitro [30]-[32], but their mechanisms of action have yet to be identified. A common mechanism of NO inhibition is not likely among these fungi as the activity appears to be cell autonomous in some species but not others, but in no case has the chemical moiety mediating the inhibition been identified. Further investigations will be necessary to determine the exact identity of these NO inhibitory compounds.

## Supporting Information

Figure S1 **Arginine biosynthesis is dispensable for NO suppression by** ***Candida albicans*** **.** Exponentially growing wildtype (SC5314), *arg1Δ* (JRC12), and *arg1*Δ + *ARG1* complemented (JRC29) fungal cells were co-cultured with LPS/IFNγ-stimulated RAW264.7 mouse macrophages at the indicated *C. albicans*:macrophage ratio. Supernatants were collected following ∼24 hours of co-culture, and nitrite levels were determined using the Greiss Reagent.
(TIF)
