# Supplementary figures and images for: Candida albicans Suppresses Nitric Oxide Generation from Macrophages via a Secreted Molecule
Source: PLoS One. 2014 Apr 22;9(4):e96203. doi: 10.1371/journal.pone.0096203 (PMC3995984; doi:10.1371/journal.pone.0096203)

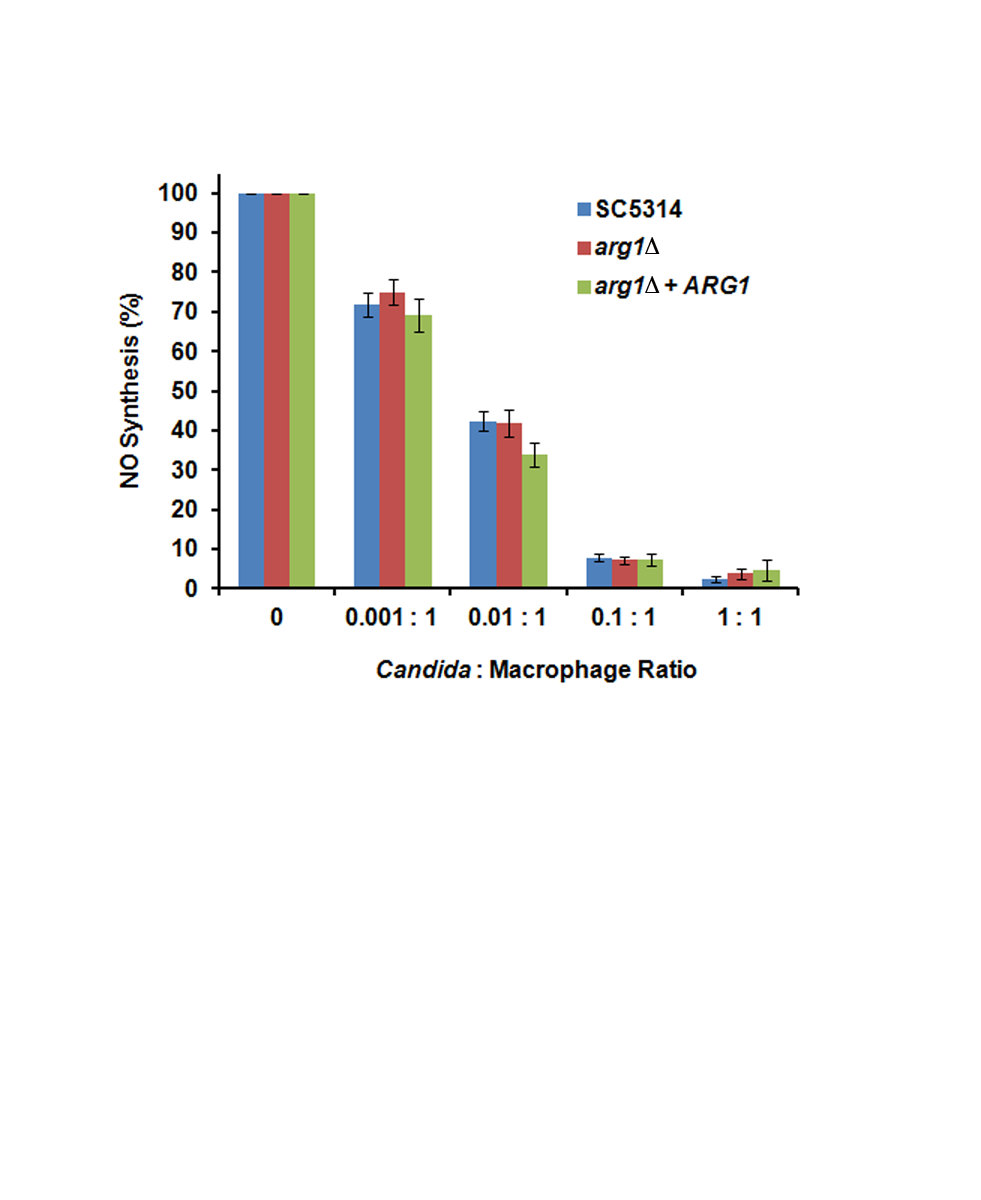

Supplement: Figure S1 — Arginine biosynthesis is dispensable for NO suppression by Candida albicans . Exponentially growing wildtype (SC5314), arg1Δ (JRC12), and arg1Δ + ARG1 complemented (JRC29) fungal cells were co-cultured with LPS/IFNγ-stimulated RAW264.7 mouse macrophages at the indicated C. albicans:macrophage ratio. Supernatants were collected following ∼24 hours of co-culture, and nitrite levels were determined using the Greiss Reagent. (TIF) [file pone.0096203.s001.tif]
